# Investigation of the Size Effects on the Elongation of Ti-2.5Al-1.5Mn Foils with Digital Image Correlation Method

**DOI:** 10.3390/ma14237353

**Published:** 2021-11-30

**Authors:** Chunju Wang, Weiwei Zhang, Zhenwu Ma

**Affiliations:** 1 Robotics and Microsystems Center, School of Mechanical and Electrical Engineering, Soochow University, Suzhou 215131, China; cjwang@suda.edu.cn; 2 Institute of Electronic Engineering, China Academy of Engineering Physics, Mianyang 621999, China; zhangweiwei0509103@163.com; 3 College of Mechanical Engineering, Suzhou University of Science and Technology, Suzhou 215009, China

**Keywords:** foil forming, size effect, digital image correlation, concentrated deformation, critical t/d value

## Abstract

The increasing demand for parts with a large specific surface area such as fuel panels has put forward higher requirements for the plasticity of foils. However, the deformation characteristics of foils is hard to be illustrated in-depth due to their very short deformation process. In this paper, the digital image correlation method was applied to investigate the influence of size effect on the elongation of Ti-2.5Al-1.5Mn foils. The results showed that the elongation of Ti-2.5Al-1.5Mn foils increased with the decrease in the ratio of thickness-to-grain diameter (t/d value). Then, the macro deformation distribution of foils was analyzed, combined with their microstructure characteristics, and it was found that the increasing influence of individual grain heterogeneity leads to the earlier formation of a concentrated deformation zone, which changes the deformation mode of foils. The concentrated deformation increases with the decrease in t/d value, thus dominating the trend of the foil elongation. Furthermore, the homogeneous deformation and concentrated deformation can be divided into two different zones by a certain critical t/d value. These results provide a basis for understanding and further exploration of the deformation behavior of titanium foils.

## 1. Introduction

With the miniaturization trend of mechanical equipment, the requirements for micro-parts are increasing significantly [1,2,3,4,5]. Among many manufacturing processes for micro-parts, the foil-forming process has attracted the most attention due to the high production efficiency and material utilization, endowing it with competitive advantage in manufacturing parts with a large specific surface area such as fuel panels and corrugated plates [6,7]. However, as the foil thickness decreases continuously, the deformation behavior of foils exhibits a strong dependence on dimension parameters, resulting in the size effects. The appearance of the size effects leads to the deterioration of plastic properties of foils, implying in-depth analyses on the deformation characteristics of foils.

The elongation is an important index to measure the plasticity of foils. In recent years, extensive studies have been devoted to the influence of size effects on the foil elongation. In general, they can be divided into two major subsets: the thickness effect and the grain size effect. The thickness effect on the elongation of CuZn20 foils has been studied via the tensile experiment, showing that the elongation decreases with the decrease in foil thickness [8]. This result can be explicated by the surface grain theory, in which the surface grain acts as a weakening factor during the deformation due to a lack of constraint of the grain boundaries [9,10]. Therefore, as the foil thickness reduces, the proportion of surface grains increases, so the fracture strength of the foil decreases, leading to less elongation. Similar phenomena have also been observed in the CuZn15 foils, CuZn36 foils, aluminum foils, etc. [11,12,13]. On the other hand, the effect of grain size on deformation behavior has been studied with pure copper foils and increased elongation, with the decrease in grain size having been demonstrated [14]. It can be explained by the fact that the grain boundary area is larger in materials with more small grains, so the distance of the crack propagation is longer before fracture, contributing to more plastic deformation of materials [15]. Consistent experimental results have been reported on copper alloy foils, stainless steel 304 foils, etc. [16,17].

To represent the interactive effects between the thickness effect and the grain size effect, the ratio of thickness-to-grain diameter (t/d value) has been proposed and applied widely, because it can interpret the experimental phenomenon of smaller sample size results in weaker deformation behavior [18,19,20]. However, as the foil thickness decreases to an order of magnitude of grain size, the heterogeneity of individual grains will dominate the deformation behavior of foils. Therefore, the individual grain heterogeneity should be taken into account when analyzing the deformation behavior of foils [21,22,23].

Taken together, most previous studies focus on the influence mechanism of size effects on the foil elongation from the perspective of grain numbers or surface grains, but there is a relative lack of research on the foil macroscopic deformation characteristics. In fact, the explicit deformation mode of a certain foil depends on comprehensively analyzing its macro and micro deformation characteristics, contributing to the illustration of the influence mechanism of size effects on the foil elongation. However, the deformation process of foils is very short, making it difficult to investigate its macroscopic deformation characteristics. The digital image correlation (DIC) technology is an optical measurement based on the calibration information of digital images. DIC has the advantages of non-contact, real-time, full-field, and local strain measurement. Therefore, it is an effective and accurate technology for studying lightweight bio-inspired composite materials [24] and measuring the full-field high-temperature thermal strain [25] and the strain in the foil deformation process. The Ti-2.5Al-1.5Mn foil is a kind of popular foil in the fuel panels and micro aircraft fields, but there is still a lack of in-depth study on its size effects during the micro-forming process. In this work, the macro and micro deformation characteristics of Ti-2.5Al-1.5Mn foil were first studied with the DIC technology. Then, the influence mechanism of size effects on the elongation of Ti-2.5Al-1.5Mn foils was explored. This study would provide a new perspective for understanding the influence of size effects on the deformation characteristics of foils.

## 2. Materials and Methods

**Figure 1:**
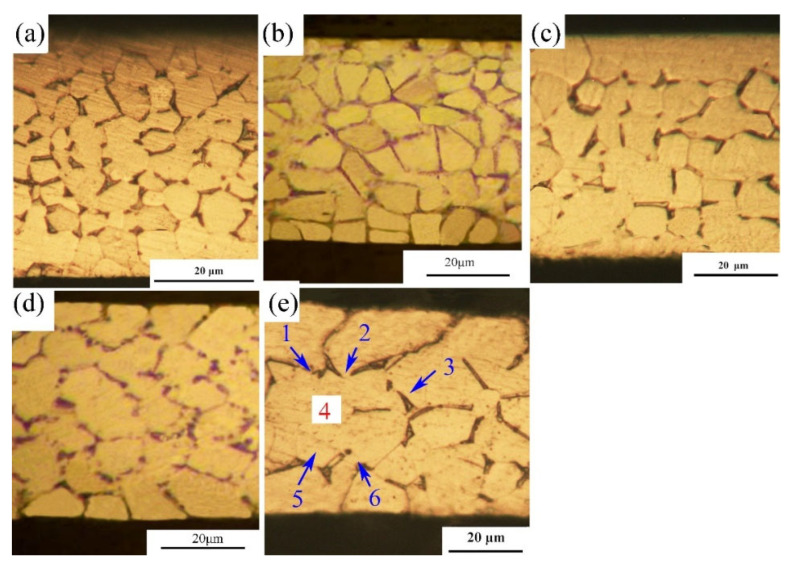
Microstructures of Ti-2.5Al-1.5Mn foils: (**a**) t/d = 13.43, (**b**) t/d = 9.37, (**c**) t/d = 7.68, (**d**) t/d = 5.14, (**e**) t/d = 3.32.

Ti-2.5Al-1.5Mn foils with a thickness of 50 μm were recrystallized and annealed at a vacuum of 1.5 × 10^−3^ Pa at different temperatures and times, so various grain sizes were obtained. Then, Ti-2.5Al-1.5Mn foils with specific t/d values (Table 1) were selected for study. The microstructures along thickness direction of foils were observed with an optical microscope (OM, Novel MR5000), as shown in Figure 1. Tensile specimens were processed by the Wire Electrical Discharge Machining with annealed Ti-2.5Al-1.5Mn foils.

The tensile test for various Ti-2.5Al-1.5Mn foils was conducted at room temperature by using the material testing machine (Shimadzu, AGS-X 5KN, Kyoto, Japan). The cross-head velocity was 1 mm/min, and the strain rate was less than 1 × 10^−3^ s^−1^. The initial measuring length and width for all specimens were kept constant as 50 and 12.5 mm, respectively. Six specimens were tested for each group.

**Figure 2:**
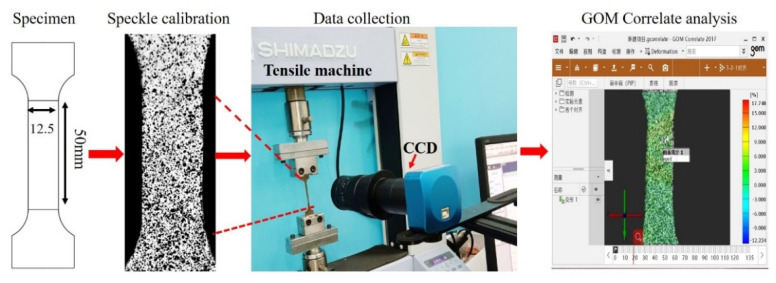
Analysis of the foil deformation process with the DIC technology.

As shown in Figure 2, the tensile deformation of Ti-2.5Al-1.5Mn foils throughout the process was captured with the DIC technology. The DIC system contains a material testing machine, a charge coupled device (CCD) camera with a 1/2-inch CMOS sensor and a 2.2 × 2.2 μm pixel size (Saga, SJ-UH500), and the GOM Correlate software. The measurement error in the DIC was ±0.01 pixels, so the influence of measurement error on deformation analysis can be neglected. The data acquisition scheme is as follows:

(1) First, a layer of white paint was sprayed on the specimen surface as the background color, and black speckles were evenly sprayed so achieve each 50% of black and white. Then, specimens were installed in the material testing machine.
(2) At the same time, when the material testing machine started running, the CCD camera, which was controlled by the GOM Correlate software, collected specimen images at the frequency of 1 image per second.
(3) The changes in marked speckle information were calculated via the GOM Correlate software, and then, the displacement and strain field information were obtained.

## 3. Results and Discussion

**Figure 3:**
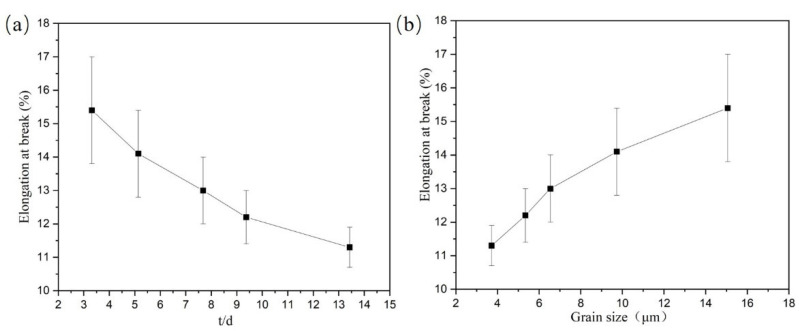
The variation of the elongation of Ti-2.5Al-1.5Mn: (**a**) with t/d value, (**b**) with grain size.

First, the elongation at the break of Ti-2.5Al-1.5Mn foils was evaluated with respect to the t/d value referring to the current size effect analysis method. As shown in Figure 3a, the foil elongation increased with the decrease in the t/d value. When the t/d value decreased from 13.43 to 3.32, the elongation of foils increased by 4.3%. This result cannot be explicated by the surface grain theory [9,10,11,12,13].

Due to the thickness of foil specimens being constant at 50 μm, the elongation of Ti-2.5Al-1.5Mn foils was evaluated with respect to the grain size. According to existing research results, for most of materials, the larger the grain size, the smaller the material elongation is [14,15,16,17]. However, as shown in Figure 3b, the elongation of Ti-2.5Al-1.5Mn foils increased as the grain size increased.

**Figure 4:**
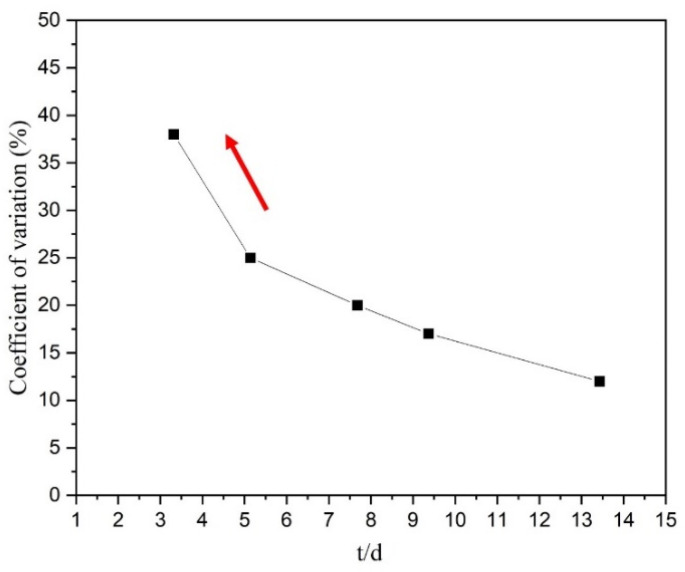
The change in C.V (*δ*) with the t/d value.

Furthermore, the scatter of the elongation between different Ti-2.5Al-1.5Mn foil specimens in the same experiment was investigated. In order to quantitatively study the scatter level of the foil elongation, the coefficient of variation (C.V) value of elongation was introduced. As shown in Figure 4, the C.V value of elongation increased with the decrease in t/d value, and the degree of this increase intensified markedly, indicating that the uniformity of the elongation of Ti-2.5Al-1.5Mn foils was becoming worse as the t/d value reduced. From the microstructures of various foils (Figure 1), the number of grains in the deformation area of the foil decreased as the annealing temperature increased. Since during the deformation process, surface grains were very easy to deform, the properties of internal grains played a significant role in the foil deformation behavior. The microhardness test for individual grains in the deformation area of foils indicated that the hardness distribution of the foils is disorderly, and the hardness distribution of foils exhibits block characteristics, which means that the hard- and soft-oriented grains are randomly distributed in the deformation area of the foil [8]. Because of the heterogeneity of individual grain properties, that is, different orientation and shape properties, the influence of individual grain heterogeneity was stronger for foils with fewer internal grains [20,21]. As shown in Figure 1e, the foil specimen with the smallest t/d value only owned three grains in the thickness direction, so its entire deformation behavior heavily depended on the deformation characteristics of individual internal grains, ultimately leading to the poor consistency of foil elongation. The drastically increased scatter behavior indicated that individual grain heterogeneity could be a critical starting point to explore the mechanism of size effects on the elongation of Ti-2.5Al-1.5Mn foils.

Compared with hard-oriented grains, soft-oriented grains tend to deform easily due to their crystal structure characteristics. It is known that the deformation performs in the manner of minimizing energy consumption. The specimen with fewer grains is prone to having a section with a large proportion of soft grains [26,27]. Therefore, a concentrated deformation zone is easy to form if the number of grains in the thickness direction is less than three. As shown in Figure 1e, if the internal grain 4 is a soft-oriented grain, the grains 1, 2, 3, 5, and 6 will deform in coordination with grain 4 in all directions to form a concentrated deformation zone. Once this concentrated deformation zone is developed, it will lead to the occurrence and concentration of remarkable deformation. Next, the DIC method is applied to study the influence of the concentrated deformation zone on the deformation behavior of foils.

**Figure 5:**
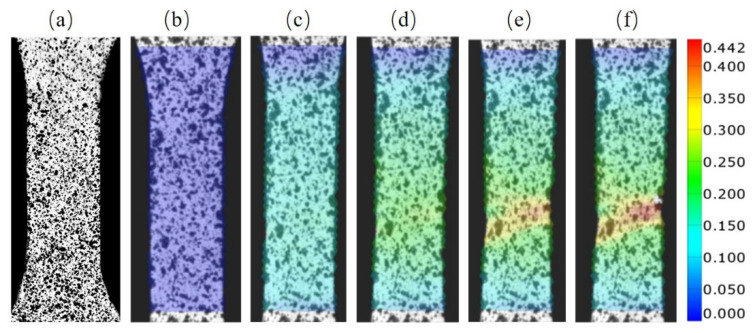
A typical equivalent strain profile of foils at different stages. (**a**) Original state, (**b**,**c**) homogeneous deformation stage, (**d**) transition of deformation stage, (**e**,**f**) concentrated deformation stage.

A typical equivalent strain profiles during the deformation process of foils were collected with the DIC method, as shown in Figure 5. The original state of speckle calibration is shown in Figure 5a. Next, in Figure 5b,c, the overall strain distribution is relatively uniform, indicating that the foil is dominated by the homogeneous deformation throughout the region at this stage. In Figure 5d, the strain distribution of the foil appears partitioned, suggesting the occurrence of deformation stage transition. In Figure 5e,f, the strain distribution exhibits the characteristics of blockiness and concentration, while the rest of the regions are basically not deformed, which proves that local deformation is prominent at this time. The graphics of each specimen in each deformation stage corresponded to its engineering strain.

**Figure 6:**
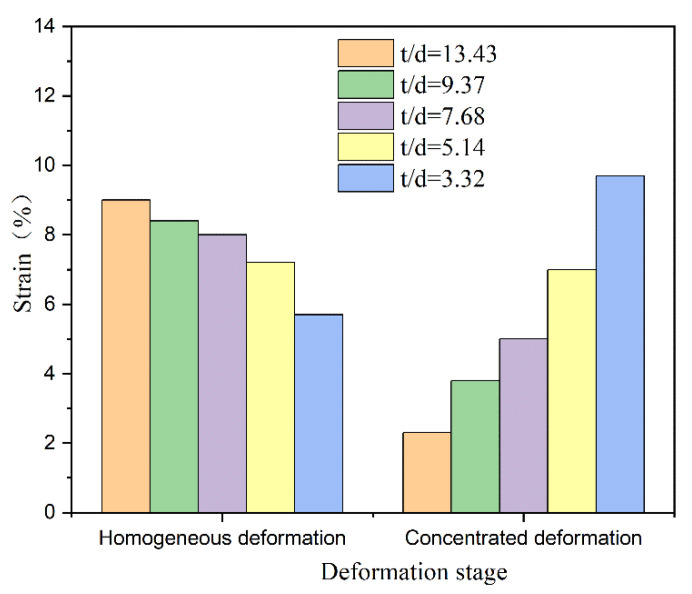
The homogeneous deformation and the concentrated deformation of various Ti-2.5Al-1.5Mn foils.

Therefore, combining the formation of concentrated deformation zone and the collected strain distribution characteristics with the DIC method, the deformation process of foils is further subdivided into the homogeneous deformation stage and the concentrated deformation stage. The critical point is set to the beginning of strain partition in this research. Accordingly, the foil deformation amount can be divided into the homogeneous deformation amount and the concentrated deformation amount. As shown in Figure 6, in the homogeneous deformation stage, the homogeneous deformation amount of foil specimens decreases with the decrease in t/d value, while in the concentrated deformation stage, the concentrated deformation amount of foil specimens increases rapidly with the decrease in t/d value. After calculating the difference values of homogeneous deformation amount and concentrated deformation amount between foils with adjacent t/d values, it can be seen that when the t/d value decreases from 5.14 to 3.32, the homogeneous deformation amount and concentrated deformation of foils amount both change markedly, suggesting the enhanced influence of concentrated deformation zone at this time. By comparing the variation degree of homogeneous deformation amount and concentrated deformation amount, it can be found that as the t/d value decreases, the increase in concentrated deformation amount is greater than the decrease in homogeneous deformation amount. Therefore, the sum of homogeneous deformation amount and concentrated deformation amount is greater for the foil specimen with a small t/d value. This is the reason why the elongation of Ti-2.5Al-1.5Mn foils increases with the decrease in the t/d value in this experiment.

Ti-2.5Al-1.5Mn foil is a close-packed hexagonal lattice metal, and there are only three slip systems that can be activated at room temperature, so its deformation behavior is very sensitive to the change in deformation state. For the foil specimen with a large t/d value, the concentrated deformation zone is not easy to form because there are numerous grains inside the deformation zone [8,17]. After the deformation begins, the movable dislocations in the soft-oriented grains first produce slip stacking, and then, the dislocation slip is transferred to other grains easily due to the coordination effect between grains. In other words, both hard-oriented and soft-oriented grains in the deformation area are involved in the deformation, gradually producing the dislocation pile-up and deformation hardening [17,18], until forming the concentrated deformation zone. Subsequently, the foil enters the concentrated deformation stage. Since these grains have already produced severe dislocation stacking and deformation hardening in the homogeneous deformation stage, the amount of further deformation that can be generated in the concentrated deformation stage is very small. Finally, the foil quickly reaches the limit and fractures in the concentrated deformation zone. Therefore, the foil with a large t/d value has a large amount of homogeneous deformation but a small amount of concentrated deformation. 

For the specimen with a small t/d value, the concentrated deformation zone is easy to form because there are a few grains in the deformation area [17]. After the deformation begins, the movable dislocations in soft-oriented grains produce slip stacking. Due to the poor coordination between grains, it is difficult to transfer the dislocation slip in soft-oriented grains to adjacent grains [27,28], resulting in the premature formation of concentrated deformation zone in this process. Subsequently, the foil quickly enters the concentrated deformation stage. Because most grains only deform slightly or do not deform in the homogeneous deformation stage, they still have a strong ability to further deform. In addition, the larger the individual grain size, the larger the area of concentrated deformation zone, which is composed of core soft-oriented grains and adjacent multiple grains, so greater deformation can be generated. This is the reason why Ti-2.5Al-1.5Mn foils that own smaller t/d value and larger grain size can produce a greater concentrated deformation amount.

**Figure 7:**
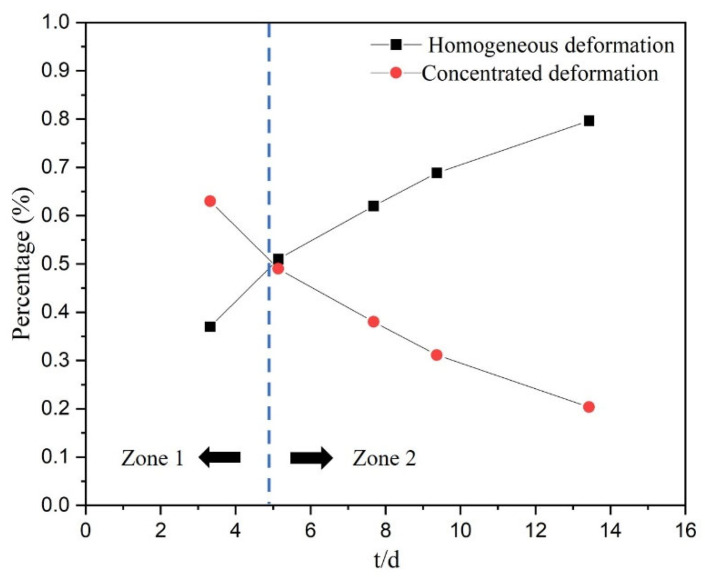
The percentage of homogeneous deformation amount and concentrated deformation amount of various Ti-2.5Al-1.5Mn foils.

The percentages of homogeneous deformation and concentrated deformation of Ti-2.5Al-1.5Mn foils with various t/d values were calculated and summarized in Figure 7. The results showed that the proportion of homogeneous deformation and concentrated deformation changes in significantly different trends, resulting in the formation of two zones, which are divided by a critical t/d value. For Ti-2.5Al-1.5Mn foils, this critical value is close to 5. In zone 2, the homogeneous deformation accounts for a large proportion and dominates the variation trend of the foil elongation. Subsequently, the proportion of concentrated deformation increases as the t/d value decreases. In zone 1, the concentrated deformation accounts for a large proportion and dominates the variation trend of the foil elongation. Due to the randomness of the position and deformation ability of the concentrated deformation zone, the uniformity of parts manufactured by using foils with the t/d value in zone 1 is poor. Taken together, it can be concluded that the plasticity of the Ti-2.5Al-1.5Mn foil with a thickness of 50 μm can be elevated by increasing its grain size, so as to produce parts with large specific surface area. However, foils with the t/d value in zone 1 should be avoided for manufacturing parts that require high forming uniformity.

## 4. Conclusions

In this work, the influence mechanism of size effect on the elongation of Ti-2.5Al-1.5Mn foil with a close-packed hexagonal crystal structure was studied with the DIC method, and the following conclusions can be drawn:

(1) The influence of individual grain heterogeneity increases during the deformation process, which results in the premature formation of concentrated deformation zones, contributing to the increased role of concentrated deformation in the foil elongation.
(2) The proportion of homogeneous deformation amount and concentrated deformation amount can be divided into two different zones by a certain critical t/d value, which is near 5 for Ti-2.5Al-1.5Mn foils.
(3) It is feasible to increase the plasticity of Ti-2.5Al-1.5Mn foils with a thickness of 50 μm by increasing the grain size, but foils with the t/d value in zone 1 should be avoided for forming parts that require high uniformity.

## Figures and Tables

**Table 1:** Annealing parameters of Ti-2.5Al-1.5Mn foils.

Number	Annealing Parameters	Grain Size d (μm)	t/d
a	700 °C, 1.5 h	3.72 ± 1.23	13.43
b	720 °C, 2.5 h	5.34 ± 2.13	9.37
c	750 °C, 3.5 h	6.52 ± 2.46	7.68
d	800 °C, 3.5 h	9.73 ± 3.15	5.14
e	850 °C, 7 h	15.06 ± 4.67	3.32

## Data Availability

The data cannot be shared because it also belongs to an ongoing research.
